# Correction: Babault et al. Usefulness of Surface Electromyography Complexity Analyses to Assess the Effects of Warm-Up and Stretching during Maximal and Sub-Maximal Hamstring Contractions: A Cross-Over, Randomized, Single-Blind Trial. *Biology* 2022, *11*, 1337

**DOI:** 10.3390/biology12020286

**Published:** 2023-02-10

**Authors:** Nicolas Babault, Marion Hitier, Carole Cometti

**Affiliations:** 1 INSERM UMR1093-CAPS, UFR des Sciences du Sport, Université de Bourgogne, F-21000 Dijon, France; 2 Centre d’Expertise de la Performance, UFR des Sciences du Sport, Université de Bourgogne, F-21000 Dijon, France; 3 Chiropractor, Private Practice, F-21000 Dijon, France

## Text Correction

There was an error in the original publication [1]. The description of the neurodynamic nerve gliding technique was incorrect. A correction has been made to *2.3. Experimental Sessions*, end of second paragraph:

“A dorsiflexion was applied in this position. Then, the volunteer performed a cervical extension concomitantly with the release of the ankle toward a neutral position. Subsequently, a cervical flexion was again performed with a concomitant dorsiflexion” should be replaced by “Then, the volunteer performed cervical extension concomitantly with dorsiflexion. Subsequently, cervical flexion was performed again with concomitant plantarflexion”.

The authors state that the scientific conclusions are unaffected. This correction was approved by the Academic Editor. The original publication has also been updated.
